# Evidence of pseudoprogression in patients treated with PD1/PDL1 antibodies across tumor types

**DOI:** 10.1002/cam4.2797

**Published:** 2020-02-19

**Authors:** Patricia Martin‐Romano, Eduardo Castanon, Samy Ammari, Stéphane Champiat, Antoine Hollebecque, Sophie Postel‐Vinay, Capucine Baldini, Andrea Varga, Jean Marie Michot, Perrine Vuagnat, Aurélien Marabelle, Jean‐Charles Soria, Charles Ferté, Christophe Massard

**Affiliations:** ^1^ Drug Development Department (DITEP) Gustave Roussy Saclay University of Paris Villejuif France; ^2^ Oncology Department Clínica Universidad de Navarra Madrid Spain; ^3^ Department of Radiology Gustave Roussy Cancer Campus Villejuif France; ^4^ INSERM VILLEJUIF France

**Keywords:** Immune checkpoint inhibitor, pseudoprogression, response evaluation criteria in solid tumors, treatment beyond progression

## Abstract

**Background:**

PD(L)1 antibodies (anti‐PD(L)‐1) have been a major breakthrough in several types of cancer. Novel patterns of response and progression have been described with anti‐PD(L)‐1. We aimed at characterizing pseudoprogression (PSPD) among patients with various solid tumor types treated by anti‐PD(L)‐1.

**Methods:**

All consecutive patients (pts) enrolled in phase 1 trials with advanced solid tumors and lymphomas treated in phase I clinical trials evaluating monotherapy by anti‐PD(L)‐1 at *Gustave Roussy* were analyzed. We aimed to assess prevalence and outcome of PSPD across tumor types. We also intended to describe potential clinical and pathological factors associated with PSPD.

**Results:**

A total of 169 patients treated with anti‐PD(L)‐1 were included in the study. Most frequent tumor types included melanoma (n = 57) and non‐small cell lung cancer (n = 19). At first tumor evaluation 77 patients (46%) presented with immune unconfirmed progressive disease. Six patients (8%) experienced PSPD: 2 patients with partial response; 4 patients with stable disease. Increase in target lesions in the first CT‐scan was more frequently associated to PSPD (67% vs 33%; *P* = .04). Patients with a PSPD had a superior survival when compared to patients progressing (median OS: 10.7 months vs 8.7 months; *P* = .07).

**Conclusions:**

A small subset of PSPD patients may experience response after an initial progression. Assessment of the current strategy for immune‐related response evaluations may require further attention.

## Graphical abstract

PD(L)1 antibodies (anti‐PD(L)‐1) have been a major breakthrough in several types of cancer. Novel patterns of response and progression have been described with these therapies. We aimed at characterizing pseudoprogression (PSPD) among patients with various solid tumor types treated by anti‐PD(L)‐1

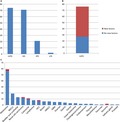

## 1 BACKGROUND

Cancer therapeutics targeting the immune system disrupted the landscape of oncology in recent years.^1^, ^2^, ^3^, ^4^ To evade the immune system, cancer cells develop a series of immunosuppressive mechanisms, including up and downregulating functional pathways favoring tumor tolerance and T‐cell anergy.^5^ The Programmed Death (PD)‐1/PD Ligand‐1 (PD‐L1) pathway is one of such critical component of tumor‐mediated immunosuppression.^6^ In this setting, the administration of antibodies targeting PD‐1 and PD‐L1 demonstrated substantial clinical benefit in patients with advanced or metastatic cancer.^1^, ^4^

The novel mechanism of action of these antibodies is associated with specific adverse events (eg, immune‐related thyroiditis, pneumonitis, or colitis, etc) along with novel patterns of response such as profound and durable responses, pseudoprogression (PSPD) and hyperprogressive disease (HPD).^1^, ^7^, ^8^ Such findings have recently led to the development of immune Response Evaluation Criteria in Solid Tumors (iRECIST) that manage Pseudoprogression (PSPD) by introducing the notion of immune unconfirmed and confirmed progressive disease (iUPD and iCPD, respectively).^9^ PSPD is defined as an initial disease progression by RECIST followed by a subsequent response. Hypotheses suggest PSPD could be related to a transient infiltration of lymphocytes into the tumor and its stroma, preceding the tumor shrinkage.^6^ Several studies evaluating immune‐related responses in patients with advanced melanoma observed PSPD in 5%‐7% of patients receiving anti‐PD(L)1.^10^, ^11^ Despite several case reports in the literature most of them including melanoma patients, the prevalence and the outcome of PSPD across solid tumors remains below 10%.^8^, ^11^, ^12^, ^13^, ^14^, ^15^ Also, to predict this transient‐progression’ or PSPD remains a challenge at bedside. The establishment of predictors of PSPD seems an important task for health care providers, given the negative consequences of either promptly discontinuing a potentially effective or maintaining an ineffective drug beyond disease progression.

These issues appear more and more prominent given the advent of immunotherapies as the new standard therapy in many cancer types. This study aimed to: (a) first, evaluate the prevalence and the outcome of PSPD across tumor types; (b) second, describe potential clinical and pathological factors associated with PSPD.

## 2 METHODS

### 2.1 Patients

All consecutive patients enrolled in phase 1 trials with advanced solid tumors and lymphomas treated in phase I clinical trials evaluating monotherapy by anti‐PD(L)‐1 at *Gustave Roussy* were analyzed. PD‐L1 positive tumors were assessed as per protocol.

### 2.2 Definition of PSPD

Antitumor Response to anti‐PD(L)‐1 was assessed according to iRECIST criteria including immune complete response (iCR), partial response (iPR), stable disease (iSD), iUPD and iCPD.^9^ Briefly, PSPD is defined as an initial progressive disease defined by an increase in the size of lesions, or the visualization of new lesions, followed by a durable response. According to iRECIST criteria, an initial progressive disease is established by as unconfirmed progressive disease (iUPD), which requires a confirmation computerized tomography scanner (CT scan) 4 to 8 weeks later. The second assessment classifies the progression as confirmed progressive disease (iCPD) by a further increase in target or new target lesions (≥5 mm in sum of measures), further increase in nontarget or new nontarget lesions, or an increase in the number of new lesions. In our cohort of patients, PSPS was established in patients with iUPD and further response considered as iCR, iPR, and iSD.

As per the different protocols, first radiological evaluation was performed per protocol 6 to 8 weeks after treatment onset, and then every 6 to 8 weeks and/or 4 to 6 in the case of iUPD. All patients had at least two radiological evaluations: baseline and first radiological examination during ICI. Disease response was based on the assessment of target lesions, nontarget lesions, and new lesions. Treatment decisions were based on investigator assessment of response per RECIST 1.1 and iRECIST. Treatment beyond progression was continued conditional to a confirmatory CT scan 4‐6 weeks after the first evidence of disease progression, as defined per protocol. All the CT scans were independently reviewed by two senior radiologists.

### 2.3 Statistical analysis

Clinical and pathological factors potentially associated with PSPD were appraised. Fisher exact test was used to assess the association between categorical variables. Hazard Ratios were estimated from Cox proportional hazard models and were adjusted to the standard clinical and pathologic prognostic factors. All the tests were two‐sided and significance was assumed if *P* < .05. Survival estimates were calculated using the Kaplan‐Meier method.^11^ Median follow up was calculated with the inverse Kaplan‐Meier method. Overall survival (OS) was determined as the time between the landmark point considered as the baseline CT scan and the death from any cause or last contact when still alive. All the analyses were carried out using STATA statistical software (version 14; STATA).

## 3 RESULTS

### 3.1 Patients’ characteristics

**Figure 1:**
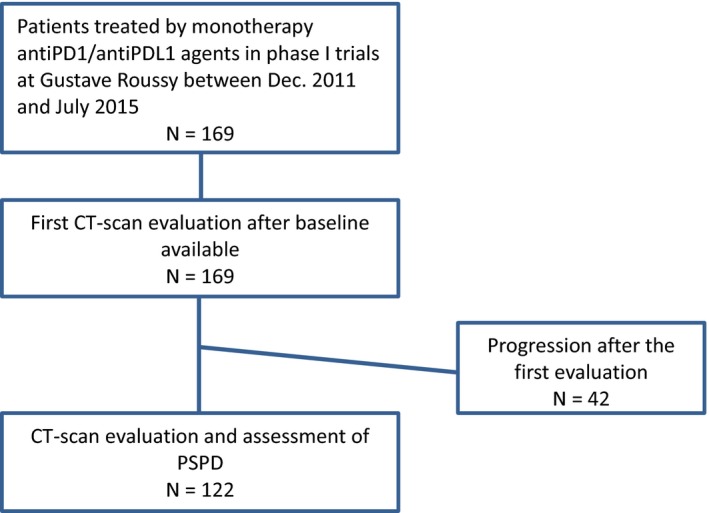
Flowchart of patients participating in the study

A total of 169 patients with solid tumors treated by anti‐PD(L)‐1 monotherapy were analyzed. All patients were enrolled in five different phase I trials at Gustave Roussy between December 2011 and July 2015. As illustrated in the flowchart of patients participating in the study (Figure 1), baseline and first evaluation CT‐scans were available in all 169 patients. Patients’ characteristics are illustrated on Table 1. The most frequent tumor types included melanoma (n = 57) and NSCLC (n = 19). Median previous lines of chemotherapy were 2 (0‐9). Patients were treated by anti PD‐1 (96 [56.5%]) and anti PD‐L1 (73 [43.5%]) monotherapy.

**Table 1:** Patient's characteristics

	Frequency (N = 169)	Proportion (%)
Tumor type		
Melanoma	57	33.7%
NSCLC	19	11.2%
Bladder and urothelial	13	7.7%
Renal cell carcinoma	12	7.1%
Colorectal	10	5.9%
Lymphoma	8	4.7%
HCC	7	4.1%
Breast	6	3.6%
HNSCC	6	3.6%
GBM	5	3%
Ovarian	5	3%
Gastric	4	2.4%
Cervix	3	1.8%
Uveal melanoma	3	1.8%
Cholangiocarcinoma	2	1.2%
Endometrium	2	1.2%
Thyroid	2	1.2%
Mesothelioma	1	0.6%
Pancreas	1	0.6%
Sarcoma	1	0.6%
Salivary gland	1	0.6%
Prostate	1	0.6%
Age (median, range)	55	20‐82
Gender		
Male	92	54.4%
Female	77	45.6%
RMH score		
0	43	25.4%
1	61	36.1%
2	55	32.5%
3	10	6%
Previous treatment lines (median, range)	2	0‐9
Type of previous treatment		
Chemotherapy	117	69.1%
Targeted therapy	95	56.2%
Immunotherapy	26	15.4%
Previous radiation	92	54.8%
Type of drug		
PD‐1 inhibitor	95	56.5%
PD‐L1 inhibitor	73	43.5%
PDL1 status		
Positive	7	4.1%
Negative	40	23.7%
NA	121	72.2%
Number of metastatic sites		
<2	70	41.4%
≥2	99	58.6%
Tumor burden estimated by RECIST 1.1 (mm, range)	75	12‐364

### 3.2 Pseudoprogression: Definition and natural history

All patients had baseline and first evaluation CT scan. The overall response rate at the first tumor evaluation and the best response rate, computed using the iRECIST criteria, are displayed in Table 2. Briefly, we observed at the first tumor evaluation: 77 patients (45.6%) with iUPD, 69 patients (40.8%) with iSD, 21 patients (12.4%) with iPR, and 2 patients (1.2%) with iCR.

**Table 2:** Patterns of response, including PSPD

	iRECIST	
	First radiological evaluation	Best overall response
iCR	2 (1.2%)	10(5.9%)
iPR	21 (12.4%)	45 (26.6%)
iSD	69 (40.8%)	43 (24.2%)
iUPD	77 (45.6%)	59 (34.9%)
iCPD	—	13 (7.7%)

**Figure 2:**
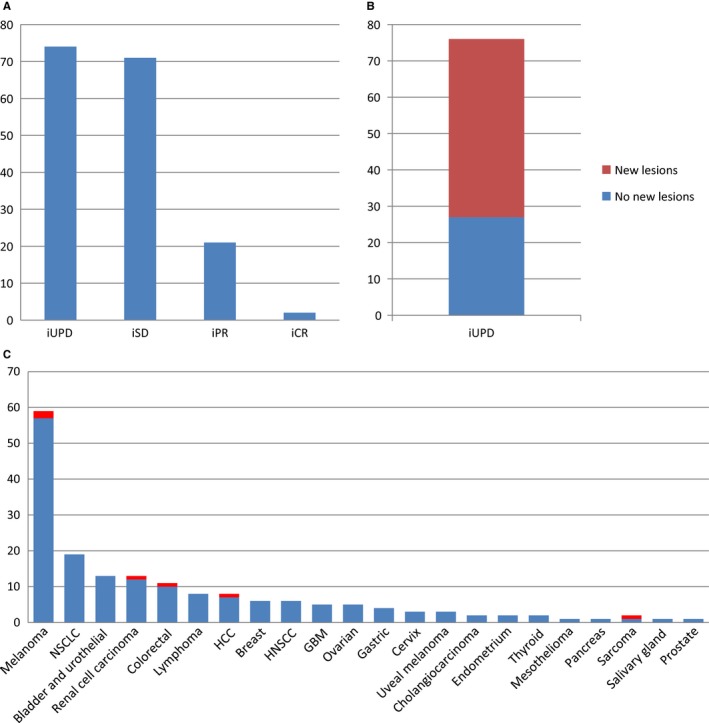
A, Distribution of the tumor responses across the iRECIST criteria at the first tumor evaluation in patients with solid tumors treated by anti‐PD‐1/PD‐L1. B, Distribution of PSPD according to best overall response in patients with solid tumors treated by anti PD‐1/PD‐L1. C, Frequency of PSPD across different tumors types (Red)

As defined above, we defined PSPD as a tumor progression (iUPD) that amended into iSD or iPR or iCR on further evaluations. Using this definition, we observed as much as 6 patients with PSPD (3.6%) out of the 169 patients. This consists in 7.8% of patients with iUPD at the first cycle (n = 76). Median time to the first CT scan showing tumor burden decrease compared with the prior CT scan was 2.2 months (range, 0.7‐3.7), and median time to the best overall response was 3.5 months (range, 0.7‐14.1). Overall, PSPD was observed in 6 patients (3.6%), with 4 (66.7%) and 2 (33.3%) patients exhibiting stable disease and partial responses respectively, after been classified as iUPD in the first evaluation. Distribution of the tumor responses across iRECIST criteria at the first tumor evaluation in patients with solid tumors treated by anti‐PD‐1/PD‐L1.) is described on Figure 2A.

On the first evaluation iUPD (n = 77) was detected due to the appearance of new lesions and increase in the size of target lesions (TL) and both of them, in 9 patients, 29 patients and 38 patients, respectively (Figure 2B).

**Figure 3:**
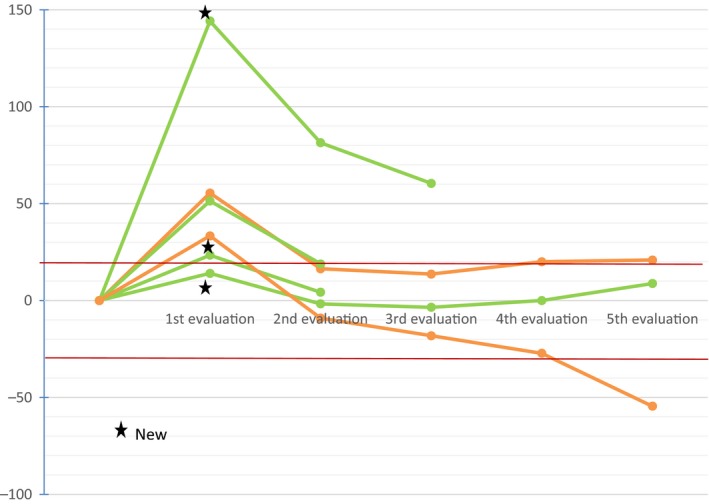
Description of the tumor burden evolution in the 6 patients with PSPD: 2 melanoma patients (orange) and 4 nonmelanoma patients (green)

Frequency of PSPD across different tumors types is illustrated on Figure 2C. PSDP occurred in 2 (1.2%) melanoma patients and 4 (2.3%) nonmelanoma patients. Phenotype PSPD included: 3 patients with increase in TL, 2 patients with new lesions and 1 patient with both of them (Table S1). Overall, two patients presented a partial response: 1 patient with a melanoma and 1 patient with colorectal cancer MSI‐H. The remaining 4 patients with a renal cell carcinoma, a sarcoma, a melanoma and a hepatocellular carcinoma displayed a stabilization of the disease. A description of the tumor burden evolution in the 6 patients with PSPD is represented in Figure 3. Median time to best response in this subset of patients was 4.3 months (2.8‐5.9).

Univariate analyses seeking for association between clinical and pathological variables and PSPD did not find any significant association (Table 2).

### 3.3 Prognostic factors for pseudoprogressive disease (LDH, AGE, RMH score, previous lines, novel lesions)

Prognostic factors for PSPD disease are represented in Table 3. No association was found between PSPD and tumor burden at baseline (estimated by the RECIST sum; *P* = .3), number of metastatic sites (*P* = 1), or Royal Marsden Hospital (RMH) prognostic score (*P* = .85). Similarly tumor type did not show a significant association with a superior likelihood of achieving PSPD (*P* = 1).

**Table 3:** Patient characteristics and association between PSPD and clinical variables (univariate analysis)

	All patients (n = 169)	Non‐PSPD (n = 163)	PSPD (n = 6)	*P* (Fisher exact test)
RMH score				
0	43 (25%)	38 (90.5%)	2 (9.5%)	0.85
1	61 (36.3%)	55 (90.2%)	2 (9.8%)	
2	55 (32.7%)	52 (94.5%)	2 (5.5%)	
3	10 (6%)	10 (100%)	0	
Tumor type				
Melanoma	57 (33.9%)	55 (96.5%)	2 (3.5%)	1.00
Non Melanoma	112 (66.1%)	108 (96.4%)	4 (3.6%)	
Gender				
Male	91 (54.2%)	86 (96.6%)	5 (3.4%)	0.219
Female	78 (45.8%)	77 (98.7%)	1 (3.9%)	
Number of previous lines				
0	21 (12.5%)	20 (95.2%)	1 (4.8%)	0.5
1	33 (19%)	31 (93.9%)	2 (6.1%)	
2	48 (28.6%)	47 (97.9%)	1 (2.1%)	
≥3	67 (39.9%)	65 (97%)	2 (3%)	
Previous chemotherapy				
Yes	117 (69.1%)	93 (97.9%)	2(2.1%)	0.07
No	52 (30.9%)	70 (94.6%)	4(5.4%)	
Previous targeted therapy				
Yes	95 (56.6%)	92 (96.7%)	3 (3.3%)	1.00
No	74 (43.5%)	71 (95.9%)	3 (4.1%)	
Previous immunotherapy				
Yes	26 (15.5%)	24 (92.3%)	2 (7.7%)	0.23
No	143 (84.5%)	139 (97.2%)	4 (2.8%)	
Previous radiation therapy				
Yes	93 (56.5%)	91 (93.5%)	2 (2.2%)	0.69
No	76 (43.5%)	72 (94.7%)	4 (5.3%)	
Type of immunotherapy				
PD‐1 inhibitor	95 (56.5%)	93 (97.9%)	2(2.1%)	0.41
PD‐L1 inhibitor	74 (42.9%)	68 (91.9%)	6 (8.1%)	
Age (median, range)	55 (20‐82)	55 (20‐82)	62 (22‐82)	0.17
Baseline LDH (UI/L)	207 (9‐1998)	207 (94‐1998)	190 (9‐741)	0.49
Baseline Fibrinogen (g/L)	4.8 (2.4‐9.6)	4.8 (2.4‐2.6)	4.3 (3‐7.9)	0.99
Baseline Albumin (g/L)	36 (20‐61)	36 (20‐61)	38 (30‐41)	0.35
Tumor burden estimated by RECIST 1.1 (mm, range)	75.5 (12‐ 364)	73 (12‐364)	62 (13‐140)	0.30
LIVER METS				
No	105 (%)	103 (98.1%)	2 (1.9%)	0.20
Yes	64 (%)	60 (93.8%)	4 (6.2%)	
Number of metastatic sites				
<2	70 (41.7%)	65 (%)	1 (%)	1
≥2	9 (58.3%)	90 (%)	5 (%)	
Appearance of new lesions at E1 (iUPD = 76)				
Yes	48 (63.2%)	45 (95.7%)	3 (6.9%)	0.76
No	28 (36.8%)	25 (89.3%)	3 (10.7%)	
Increase target lesions (iUPD = 76)				
Yes	64 (54.4%)	60 (93.8%)	4 (6.2%)	0.04
No	12 (45.6%)	10 (83.3%)	2 (16.7%)	
Increase nontarget lesions (iUPD = 76)				
Yes	43 (%)	42 (97.7%)	1 (2.4%)	0.97
No	33 (%)	28 (84.8%)	5 (15.2%)	

Abbreviations: CRP, C‐reactive protein; LDH, lactate dehydrogenase.

* For continuous variables *P* value was calculated from Wilcoxon Rank Sum Test.

We examined the potential influence of previous therapies, including chemotherapy, radiation therapy, targeted therapies, and earlier immunotherapy. Results did not display association between PSPD and the number of previous lines (*P* = .49), nor previous treatment type including targeted therapy (*P* = 1), radiotherapy (*P* = .7) and immunotherapy (*P* = .41). The absence of previous conventional chemotherapy was associated with a trend for PSPD (*P* = .07).

Results did not show statistically significant differences in the rate of PSPD between patients treated with anti‐PD(L)‐1 agents (*P* = .41).

There was no difference within the baseline blood characteristics between PSPD and non‐PSPD patients at baseline such as LDH (*P* = .49), fibrinogen (*P* = 1), and albumin (*P* = .35).

The association between PSPD and changes in target and nontarget lesions was also evaluated. Patients with an increase in target lesions were more likely to present PSPD (*P* = .04). We did not observe a significant difference between PSPD status and either an increase of nontarget lesions or the appearance of new lesions.

### 3.4 Association between iRECIST response, PSPD and survival

The median follow‐up time was 46 months (95% CI, 39.7‐49.3). Median PFS and OS were 17.4 months (95% CI, 6.2‐33.9) and 20.6 months (95% CI, 15.5‐34.7), respectively.

Time to best overall response in PSPD patients was 4.3 months (range, 2.8‐5.9).

To investigate the association between PSPD and prognosis, we performed Kaplan‐Meier OS estimates (landmark survival analysis) according to the following classes: iCR, iPR, iSD, PSPD, and progressive patients (iUPD and iCPD). Patients with a PSPD had a similar OS (median OS, 10.7 months; 95% CI, 6.5‐NA) when compared to patients with patients progressing in the first CT scan (median OS, 8.7 months; 95% CI, 7.1‐11.6 [HR 0.39, 95% CI, 0.14‐1.07; *P* = .07). Median OS was not reached in patients with CR and PR, and was 23.8 months (95% CI, 12.9‐41.3) in patients with SD. The overall log‐rank test was significant (*P* < 1e‐5) as well between all groups.

## 4 DISCUSSION

After being classified as progressive disease within the first radiological assessment, there is a small subset of patients treated with anti‐PD(L)‐1 monotherapy that appears to experience tumor responses as our results demonstrate. PSPD has been recently defined as an initial disease progression either by an increase in the size of lesions, or the appearance of new lesions within the first weeks after treatment onset.^9^ Conventional criteria for tumor response assessment is also known as Response Evaluation Criteria in Solid Tumors (RECIST) have been the standardized method to evaluate tumor responses to cytotoxic agents.^16^ The development of immunotherapy has implied the development of new criteria to appraise immune‐related responses.^9^, ^17^, ^18^ Within the first evaluation, 45% of patients displayed progressive disease classified as iUPD. Among these patients classified as iUPD, 8% of patients actually presented PSPD beyond the first evaluation. Nevertheless, this patient subgroup represents a substantial challenge given the absence of standardized criteria regarding clinical management of these patients. In this regard, a prompt therapy discontinuation in these patients presenting an early progressive disease within the first evaluation could imply a risk of dismissing a potentially effective drug. Interruption of treatment should be decided depending on whether patients are experiencing a clinical benefit and in the absence of severe toxicities.

Globally, approximately 4% of our patients treated with an anti‐PD(L)‐1 agents displayed a PSPD at first evaluation. Together with previously published results, our study confirms that PSPD represents a relatively uncommon event as well a real challenge in the daily oncological practice. Our results showed that PSPD appears to be more frequent in patients experiencing an initial increase in target lesions, suggesting that PSPD should be investigated only in these patients. Biologic explanation of the increase in lesions including enlarging nodal, pulmonary or other visceral lesions has been reported as immune‐mediated sarcoid reactions with pathological reports of nonmalignant granulomas.^19^ Nevertheless, an initial increment of tumor burden along with the appearance of new lesions usually corresponds to a real disease progression and is accompanied by a clinical deterioration. To acknowledge the real impact of PSPD requires further studies in larger and prospective cohorts.

Immune checkpoint inhibitors are now approved in a wide range of tumor types, including melanoma, NSCLC, RCC, HNSCC, urothelial, and microsatellite instability‐high (MSI) cancer, although response rates are different.^14^, ^20^, ^21^, ^22^, ^23^, ^24^ Similarly, different rates of PSPD have been described depending on the tumor type. Partial responses following an initial progression have been reported in 5%‐8% of melanoma and renal cell carcinoma patients treated with anti‐PD(L)‐1.^14^, ^20^ Lower rates of radiological responses or stabilizations have been outlined in other tumor types such as NSCLC, HNSCC and urothelial carcinomas, ranging from 1% to 7% in patients also treated with anti‐PD(L)‐1 agents. Pseudoprogression has also been pointed out in patients with MSI tumors treated with anti‐PD(L)‐1 agents.^25^ Overall, this event does not exceed 10% independent of tumor type. Interestingly, the two patients presenting a PSPD in our series had a melanoma and an MSI‐high CRC. These two tumor types are well‐known as hypermutated tumors and most likely to have immune responses, although translational studies exploring PSPD underlying mechanisms and how to predict this event are lacking. Circulating Tumor DNA (ctDNA) profiles have been evaluated as an early accurate biomarker able to identify pseudoprogressors and differentiate them from true progressors.^26^, ^27^ Performing sequential ctDNA tests to patients receiving anti‐PD(L)‐1 agents could represent an alternative to dynamically monitor these patients, although further validation is needed in larger cohorts of patients.

In our series, patients who had not received a prior conventional chemotherapy tend to have more frequently a PSPD when compared to patients already treated with chemotherapy. Usually, heavily pretreated patients tend to have limited responses and these are short‐lasting. When analyzed by subgroups median survival of patients with PSPD was numerically superior to patients truly progressing (10.7 vs 8.7 months; *P* = .07). A recently published study demonstrated responder patients displayed a higher 12‐month OS rate than those with an initial increase in tumor burden (82% vs. 53%).^28^ Time to best overall response in PSPD patients ranged up to 5.9 months, with a median of 4 months. Interestingly, a similar study assessing only NSCLC patients reported tumor regression at 3 and 4 months after PSPD.^24^ Limitations of our study include its retrospective nature, heterogeneity of the patients including melanoma and nonmelanoma patients and the fact that it was developed in a single institution. Patients from our series had been previously heavily pretreated with a median of 2 previous therapy lines, including nearly 70% who had received chemotherapy and 57% targeted therapy. On the other hand, all patients from our study were treated on the basis of a phase I trial from 2011 to 2015, when most immunotherapy trials assessed anti‐PD(L)‐1 agents in the monotherapy setting. This fact implies that most patients were treated out of the scope of iRECIST criteria, that a percentage of these patients might have discontinued treatment without a confirmation of disease progression and, moreover, without the evolution of a potentially beneficial therapy. In addition and given the small number of patients included in our study, it seems clear that larger cohorts are needed to further assess the importance of immune‐related response and its impact on long‐term results.

In conclusion, PSPD is a rare phenomenon observed in less than 5% of patients receiving anti‐PD(L)‐1 therapy. These patients present a longer overall survival than patients presenting a progressive disease, although not as encouraging as patients with real responses. We have not identified any clinical or analytical factor that may predict this type of response. Further investigation is warranted in order to identify PSPD patients and avoid therapy discontinuation, since a benefit in terms of OS may be observed after.

## CONFLICT OF INTERESTS

The authors declare the following COI: Principal/sub‐Investigator of Clinical Trials for Abbvie, Agios Pharmaceuticals, Amgen, Argen‐X Bvba, Arno Therapeutics, Astex Pharmaceuticals, Astra Zeneca, Aveo, Bayer Healthcare Ag, Bbb Technologies Bv, Blueprint Medicines, Boehringer Ingelheim, Bristol Myers Squibb, Celgene Corporation, Chugai Pharmaceutical Co., Clovis Oncology, Daiichi Sankyo, Debiopharm SA, Eisai, Eli Lilly, Exelixis, Forma, Gamamabs, Genentech, Inc, Glaxosmithkline, H3 Biomedicine, Inc, Hoffmann La Roche Ag, Innate Pharma, Iris Servier, Janssen Cilag, Kyowa Kirin Pharm. Dev., Inc, Loxo Oncology, Lytix Biopharma As, Medimmune, Menarini Ricerche, Merck Sharp & Dohme Chibret, Merrimack Pharmaceuticals, Merus, Millennium Pharmaceuticals, Nanobiotix, Nektar Therapeutics, Novartis Pharma, Octimet Oncology Nv, Oncoethix, Onyx Therapeutics, Orion Pharma, Oryzon Genomics, Pfizer, Pharma Mar, Pierre Fabre, Roche, Sanofi Aventis, Taiho Pharma, Tesaro, Inc, Xencor. Research Grants from Astrazeneca, BMS, Boehringer Ingelheim, Janssen Cilag, Merck, Novartis, Pfizer, Roche, Sanofi. Non‐financial support (drug supplied) from Astrazeneca, Bayer, BMS, Boringher Ingelheim, Johnson & Johnson, Lilly, Medimmune, Merck, NH TherAGuiX, Pfizer, Roche. CM has received honoraria from Astellas, Astra Zeneca, Bayer, BeiGene, BMS, Celgene, Debiopharm, Genentech, Ipsen, Janssen, Lilly, MedImmune, MSD, Novartis, Pfizer, Roche, Sanofi, Orion; SC has received honoraria from AstraZeneca, BMS, Janssen, MSD, Novartis and Roche. A. H. has received honoraria from Amgen, Spectrum Pharmaceuticals, Lilly. Over the last 5 years, Dr Soria has received consultancy fees from AstraZeneca, Astex, Clovis, GSK, GamaMabs, Lilly, MSD, Mission Therapeutics, Merus, Pfizer, PharmaMar, Pierre Fabre, Roche/Genentech, Sanofi, Servier, Symphogen, and Takeda. Dr Soria has been a full‐time employee of AstraZeneca since September 2017. He is a shareholder of AstraZeneca and Gritstone. CF became a full‐time employee of Medimmune/AstraZeneca after this article was commissioned. AM has received honoraria from GSK, AstraZeneca, Oncovir, Merck Serono, eTheRNA, Lytix pharma, Kyowa Kirin Pharma, Bayer, Novartis, BMS, Symphogen, Genmab, Amgen, Biothera, Nektar, Pfizer, Seattle Genetics, Flexus Bio, Roche/Genentech, OSE immunotherapeutics, Transgene, Gritstone, Merck (MSD), Cerenis, Innate pharma, Protagen, Partner Therapeutics, Servier. SPV has received honoraria from Merck KGaA, AstraZeneca. EC has received honoraria from BMS, Roche, MSD and Medimmune.

## AUTHORS’ CONTRIBUTIONS

Conception and design: PMR, SA, ECA, CF, CM. Provision of study materials or patients: All authors. Collection and assembly of data: PMR, SA, ECA, CF, CM. Data analysis and interpretation: All authors. Manuscript writing: All authors. Final approval of manuscript: All authors. Accountable for all aspects of the work: All authors.

## ETHICAL STANDARDS AND CONSENT TO PARTICIPATE

All procedures followed were in accordance with the ethical standards of the responsible committee on human experimentation (institutional and national) and with the Helsinki Declaration of 1964 and later versions. Informed consent was obtained from all patients for being included in the study.

## CONSENT FOR PUBLICATION

Not applicable.

## Data Availability

The data used and/or analyzed for this study is available from the corresponding author at reasonable request.

## References

[1] Brahmer JR , Drake CG , Wollner I , et al. Phase I study of single‐agent anti‐programmed death‐1 (MDX‐1106) in refractory solid tumors: safety, clinical activity, pharmacodynamics, and immunologic correlates. J Clinic Oncol. 2010;28(19):3167‐3175. 10.1200/JCO.2009.26.7609 PMC4834717 20516446

[2] Hodi FS , O'Day SJ , McDermott DF , et al. Improved survival with ipilimumab in patients with metastatic melanoma. New Engl J Med. 2010;363(8):711‐723. 20525992 10.1056/NEJMoa1003466 PMC3549297

[3] Robert C , Thomas L , Bondarenko I , et al. Ipilimumab plus dacarbazine for previously untreated metastatic melanoma. New Engl J Med. 2011;364(26):2517‐2526. 21639810 10.1056/NEJMoa1104621

[4] Brahmer JR , Tykodi SS , Chow LQM , et al. Safety and activity of anti‐PD‐L1 antibody in patients with advanced cancer. New Engl J Med. 2012;366(26):2455‐2465. 22658128 10.1056/NEJMoa1200694 PMC3563263

[5] Schreiber RD , Old LJ , Smyth MJ . Cancer immunoediting: integrating immunity's roles in cancer suppression and promotion. Science. 2011;331(6024):1565‐1570. 21436444 10.1126/science.1203486

[6] Tumeh PC , Harview CL , Yearley JH , et al. PD‐1 blockade induces responses by inhibiting adaptive immune resistance. Nature. 2014;515(7528):568‐571. 25428505 10.1038/nature13954 PMC4246418

[7] Champiat S , Dercle L , Ammari S , et al. Hyperprogressive disease is a new pattern of progression in cancer patients treated by anti‐PD‐1/PD‐L1. Clinic Cancer Res. 2017;23(8):1920‐1928. 10.1158/1078-0432.CCR-16-1741 27827313

[8] Borcoman E , Kanjanapan Y , Champiat S , et al. Novel patterns of response under immunotherapy. Annals Oncol. 2019;30(3):385‐396. 10.1093/annonc/mdz003 30657859

[9] Seymour L , Bogaerts J , Perrone A , et al. iRECIST: guidelines for response criteria for use in trials testing immunotherapeutics. Lancet Oncol. 2017;18(3):e143‐e152. 28271869 10.1016/S1470-2045(17)30074-8 PMC5648544

[10] Hodi FS , Hwu W‐J , Kefford R , et al. Evaluation of immune‐related response criteria and RECIST v1.1 in patients with advanced melanoma treated with pembrolizumab. J Clinic Oncol. 2016;34(13):1510‐1517. 10.1200/JCO.2015.64.0391 PMC5070547 26951310

[11] Long GV , Weber JS , Larkin J , et al. Nivolumab for patients with advanced melanoma treated beyond progression: analysis of 2 phase 3 clinical trials. JAMA Oncol. 2017;3(11):1511‐1519. 28662232 10.1001/jamaoncol.2017.1588 PMC5710191

[12] George S , Motzer RJ , Hammers HJ , et al. Safety and efficacy of nivolumab in patients with metastatic renal cell carcinoma treated beyond progression: a subgroup analysis of a randomized clinical trial. JAMA Oncol. 2016;2(9):1179‐1186. 27243803 10.1001/jamaoncol.2016.0775 PMC5568541

[13] Blumenthal GM , Theoret MR , Pazdur R . Treatment beyond progression with immune checkpoint inhibitors‐known unknowns. JAMA Oncol. 2017;3(11):1473‐1474. 28662228 10.1001/jamaoncol.2017.1819

[14] Escudier B , Motzer RJ , Sharma P , et al. Treatment beyond progression in patients with advanced renal cell carcinoma treated with nivolumab in checkmate 025. Eur Urol. 2017;72(3):368‐376. 28410865 10.1016/j.eururo.2017.03.037

[15] Kazandjian D , Keegan P , Suzman DL , Pazdur R , Blumenthal GM . Characterization of outcomes in patients with metastatic non‐small cell lung cancer treated with programmed cell death protein 1 inhibitors past RECIST version 1.1‐defined disease progression in clinical trials. Semin Oncol. 2017;44(1):3‐7. 28395760 10.1053/j.seminoncol.2017.01.001

[16] Eisenhauer EA , Therasse P , Bogaerts J , et al. New response evaluation criteria in solid tumours: revised RECIST guideline (version 1.1). Eur J Cancer. 2009;45(2):228‐247. 19097774 10.1016/j.ejca.2008.10.026

[17] Wolchok JD , Hoos A , O'Day S , et al. Guidelines for the evaluation of immune therapy activity in solid tumors: immune‐related response criteria. Clinic Cancer Res. 2009;15(23):7412‐7420. 10.1158/1078-0432.CCR-09-1624 19934295

[18] Nishino M , Giobbie‐Hurder A , Gargano M , Suda M , Ramaiya NH , Hodi FS . Developing a common language for tumor response to immunotherapy: immune‐related response criteria using unidimensional measurements. Clinic Cancer Res. 2013;19(14):3936‐3943. 10.1158/1078-0432.CCR-13-0895 PMC3740724 23743568

[19] Cousin S , Toulmonde M , Kind M , et al. Pulmonary sarcoidosis induced by the anti‐PD1 monoclonal antibody pembrolizumab. Annals Oncol. 2016;27(6):1178‐1179. 10.1093/annonc/mdw125 27091806

[20] Weber JS , D'Angelo SP , Minor D , et al. Nivolumab versus chemotherapy in patients with advanced melanoma who progressed after anti‐CTLA‐4 treatment (CheckMate 037): a randomised, controlled, open‐label, phase 3 trial. Lancet Oncol. 2015;16(4):375‐384. 25795410 10.1016/S1470-2045(15)70076-8

[21] Motzer RJ , Rini BI , McDermott DF , et al. Nivolumab for metastatic renal cell carcinoma: results of a randomized phase II trial. J Clinic Oncol. 2015;33(13):1430‐1437. 10.1200/JCO.2014.59.0703 PMC4806782 25452452

[22] Sharma P , Callahan MK , Bono P , et al. Nivolumab monotherapy in recurrent metastatic urothelial carcinoma (CheckMate 032): a multicentre, open‐label, two‐stage, multi‐arm, phase 1/2 trial. Lancet Oncol. 2016;17(11):1590‐1598. 27733243 10.1016/S1470-2045(16)30496-X PMC5648054

[23] Le DT , Uram JN , Wang H , et al. PD‐1 blockade in tumors with mismatch‐repair deficiency. New Engl J Med. 2015;372(26):2509‐2520. 26028255 10.1056/NEJMoa1500596 PMC4481136

[24] Tazdait M , Mezquita L , Lahmar J , et al. Patterns of responses in metastatic NSCLC during PD‐1 or PDL‐1 inhibitor therapy: Comparison of RECIST 1.1, irRECIST and iRECIST criteria. Eur J Cancer. 1990;2018(88):38‐47. 10.1016/j.ejca.2017.10.017 29182990

[25] Chae YK , Wang S , Nimeiri H , Kalyan A , Giles FJ . Pseudoprogression in microsatellite instability‐high colorectal cancer during treatment with combination T cell mediated immunotherapy: a case report and literature review. Oncotarget. 2017;8(34):57889‐57897. 28915720 10.18632/oncotarget.18361 PMC5593692

[26] Lee JH , Long GV , Menzies AM , et al. Association between circulating tumor DNA and pseudoprogression in patients with metastatic melanoma treated with anti‐programmed cell death 1 antibodies. JAMA Oncol. 2018;4(5):717‐721. 29423503 10.1001/jamaoncol.2017.5332 PMC5885201

[27] Cabel L , Riva F , Servois V , et al. Circulating tumor DNA changes for early monitoring of anti‐PD1 immunotherapy: a proof‐of‐concept study. Annals Oncol. 2017;28(8):1996‐2001. 10.1093/annonc/mdx212 28459943

[28] Nishino M , Giobbie‐Hurder A , Manos MP , et al. Immune‐related tumor response dynamics in melanoma patients treated with pembrolizumab: identifying markers for clinical outcome and treatment decisions. Clinic Cancer Res. 2017;23(16):4671‐4679. 10.1158/1078-0432.CCR-17-0114 PMC5559305 28592629

