# Supplementary material for: Evidence of pseudoprogression in patients treated with PD1/PDL1 antibodies across tumor types
Source: Cancer Med. 2020 Feb 19;9(8):2643–52. doi: 10.1002/cam4.2797 (PMC7163099; doi:10.1002/cam4.2797)
Supplement: Supplementary file 1 [file CAM4-9-2643-s001.docx]

Supplementary Table 1. Phenotype of patients presenting PSPD

|  | **Tumor type** | **PSPD phenotype** | **Best Overall response** |
| --- | --- | --- | --- |
| Patient 1 | Melanoma | New TL | iPR |
| Patient 2 | RCC | Increase TL | iSD |
| Patient 3 | Sarcoma | Increase TL, new TL | iSD |
| Patient 4 | Melanoma | New TL | iSD |
| Patient 5 | HCC | Increase TL | iSD |
| Patient 6 | CRC | Increase TL | iPR |
